# Disseminated nocardiosis in a patient with alcoholic liver cirrhosis: a case report

**DOI:** 10.1186/s12879-023-08421-7

**Published:** 2023-07-01

**Authors:** Rewaa Alqurashi, Husam Alobida, Abdullah Albathi, Moneera Aldraihem

**Affiliations:** 1 Department of Internal Medicine, King Fahad Medical City, Riyadh, Saudi Arabia; 2 Department of Infectious Disease, King Fahad Medical City, Riyadh, Saudi Arabia; 3 Department of Radiology, King Fahad Medical City, Riyadh, Saudi Arabia; 4 Department of Neurology, King Fahad Medical City, Riyadh, Saudi Arabia

**Keywords:** Infectious diseases, Nocardia, Liver disease, Neurology

## Abstract

**Background:**

Nocardia are Gram-positive, aerobic, filamentous bacteria that can cause localized or disseminated infections. Immunocompromised patients are at a higher risk of developing Nocardia infection and further dissemination of the disease. To date, limited data have documented the relationship between nocardiosis and alcoholic liver disease.

**Case presentation:**

We report the case of a 47-year-old man with a known history of alcoholic liver cirrhosis. The patient presented to our emergency department with redness, swelling in the left eye, and diminished bilateral vision. Fundus examination of the left eye was obscured, while that of the right eye was consistent with subretinal abscess. Therefore, endogenous endophthalmitis was suspected. Imaging revealed two ring-enhancing lesions in the brain, and multiple bilateral small cystic and cavitary lung lesions. Unfortunately, the left eye eventually eviscerated due to the rapid progression of the disease. Cultures from the left eye were positive for *Nocardia farcinica*. The patient was started on imipenem, trimethoprim/sulfamethoxazole, and amikacin based on culture sensitivity. The patient’s hospitalization course was complicated by his aggressive and advanced condition, which led to his death.

**Conclusions:**

Although the patient’s condition initially improved with the recommended antibiotic regimens, it led to death owing to the patient’s advanced condition. Early detection of nocardial infection in patients with typical or atypical immunosuppressive conditions may improve overall mortality and morbidity. Liver cirrhosis disrupts cell-mediated immunity and may increase the risk of Nocardia infection.

## Background

Nocardia are Gram-positive aerobic filamentous bacteria that can cause localized (such as pulmonary and primary cutaneous nocardiosis) or disseminated infections. Nocardiosis is uncommon, and an established bacterial infection usually affects immunocompromised patients with cell-mediated abnormalities; however, one-third of patients are immunocompetent [1, 2].

Between 1995 and 2004, 765 Nocardia cases were submitted to the Centers for Disease Control and Prevention (CDC): 28% *N. nova* complex, 14% *N. brasiliensis*, 14% *N. farcinica*, and 13% *N. cyriacigeorgica* [3].

Despite treatment, nocardiosis was reported to have a high morbidity, and 23.1% of patients had a chronic disease that could not be cured with a high risk of recurrence. Many of these patients developed complications such as scarring, impaired vision, limp amputation, and residual neurological impairment. Regarding mortality, 5.7% of patients died because of complications of the disease [4].

Major risk factors for nocardial infection include typical immunosuppressive conditions, such as cancer, bone marrow or solid organ transplantation, high doses of corticosteroids, and HIV/AIDS. Other atypical conditions include diabetes, connective tissue diseases, alcoholism, and pulmonary alveolar proteinosis [5].

Immunocompromising entities such as autoimmune diseases,and lymphopenia subjects patients to a higher risk of developing disseminated nocardiosis. Soueges et al. found that patient who were infected with Nocardia farcinica were at a higher risk of dissemination [6].

The pace and course of the disease are primarily related to the patient’s immune status. In immunocompetent patients, the disease usually has a chronic course and is likely to be localized to a single area or organ. Hematogenous dissemination is more common in immunocompromised hosts and usually involves the central nervous system and skin [2].

To date, limited data have documented the relationship between nocardiosis and alcoholic liver disease. In this report, we present a case of disseminated nocardia with endogenous endophthalmitis; two ring-enhancing lesions in the brain; and multiple bilateral small, cystic, and cavitary lung lesions in a patient with a medical history of alcoholic liver cirrhosis.

## Case presentation

The patient was a 47-year-old man with a history of heavy alcohol intake over the past 4 years (3–4 drinks per day), which was complicated by alcoholic liver cirrhosis (Child–Pugh C/MELD 22), portal hypertension, and esophageal varices grade 2. He presented to our emergency department with redness and swelling in the left eye for 2 months and bilateral diminished vision, mainly in the left eye, over the previous month. The patient had no history of ocular trauma. The associated symptoms included mild right upper quadrant pain, jaundice, unintentional weight loss (approximately 22 kg in the past 3 months), pruritus, and dark-colored urine. Ophthalmic examination done upon presentation is shown in (Table 1).

**Table 1:** Ophthalmic examination upon presentation

*Ophthalmic examination*	Right	Left
External Exam	Normal	Mild eyelid swelling, severe conjunctival congestion, hyperemia
Visual acuity	Accurate perception of light	No light perception
Slit Lamp Exam		
Cornea	Clear	Clear
Anterior chamber	Deep and quite	+ 3 cells and + 2 flare
Iris	Medically dilated	Posterior synechiae
Lense	Clear	Clear
Vitreous	Clear	+ 4 cells
Fundus exam		
Macula	Large sub-retinal white to yellow abscess measuring 6 mm, extending beyond temporal arcades and cover all macula	Obscured

The initial blood analysis was as follows: complete blood count: white blood cells: 10.3 × 10^3^/µL (normal range 4.5 to 11.0 × 10^6^/μL), red blood cells: 3.5 × 10^6^/μL (normal range 4.7 to 6.1 × 10^6^/μL), hemoglobin: 11.2 g/ldl (normal range 13.2 to 16.6 g/dl), platelets: 225 × 10^3^/µL (normal range 150 to 400 × 10^3^/µL); creatinine: 64 µmol/L (normal range 53 to 97.2 µmol/L), serum sodium: 131 mmol/L (normal range 136 and 145 mmol/L), serum potassium: 4.4 mmol/L (normal range 3.6 to 5.2 mmol/L), serum magnesium: 0.74 mmol/L (normal range 0.85 to 1.10 mmol/L), corrected calcium: 2.2 mmol/L (normal range 2.2 to 2.7 mmol/L), Erythrocyte sedimentation rate: 95 mm/hr (normal range 0 to 15 mm/hr); C-reactive protein level: 92 mg/L (normal range < 3 mg/L); total bilirubin: 132 µmol/L (normal range 1.71 to 20.5 µmol/L); alkaline phosphatase: 151 U/L (normal range 44 to 147 U/L); alanine aminotransferase: 33 U/L (normal range 4 to 36 U/L); albumin: 28.7 g/L (normal range 34 to 54 g/L); and lactic acid: 1.8 mmol/L (normal range 0.5 to 2.2 mmol/L).

**Fig. 1:**
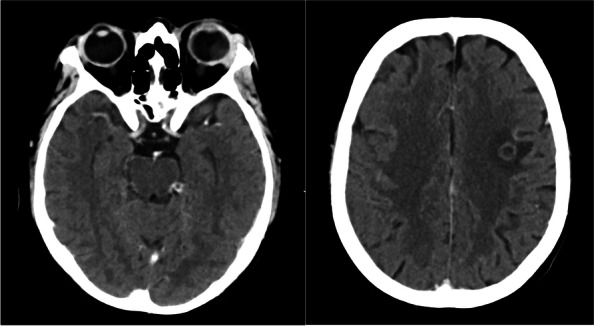
*Left image*: Axial contrast-enhanced CT scan of the orbits showing generalized scleral thickness of the left eye with heterogeneous increase in density of the vitreous content. Also seen is soft tissue swelling, orbital proptosis, peri- and post-septal orbital fat stranding plus thickening, and enhancement of the globe wall. Findings that are suggestive of endophthalmitis and subretinal abscess. Enlargement of the lacrimal gland is also noted. *Right image*: Axial section of a contrast-enhanced CT of the brain shows a ring- enhancing lesion in the left frontal lobe measuring 0.9 cm with surrounding vasogenic edema. Findings consistent with intraparenchymal abscess

**Fig. 2:**
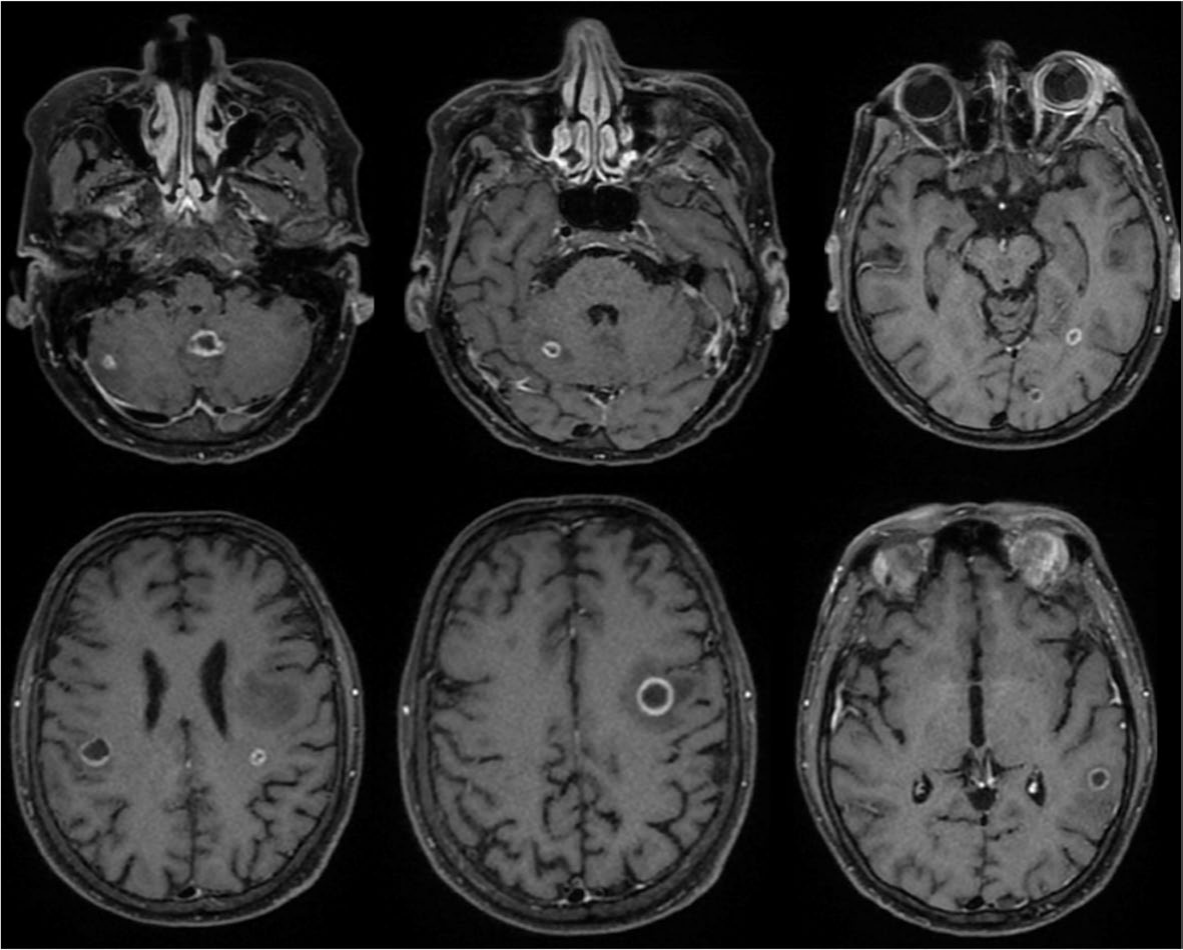
*Contrast-enhanced T1-weighted images.* Multiple supra- and infratentorial rim-enhancing lesions. Also noted surrounding vasogenic edema. Findings that are consistent with abscesses. Images on the right show enhancement of the left globe and surrounding soft tissue. Relatively hyperintense subretinal fluid is noted. Findings that are consistent with left eye endophthalmitis and subretinal abscess. The right orbit shows enhancement of the extra-conal soft tissue suggesting involvement

Computed tomography (CT) of the brain and orbits revealed two ring-enhancing lesions in the left frontal lobe along with orbital findings suggestive of left orbital endophthalmitis (Fig. 1). Contrast-enhanced magnetic resonance imaging was performed to confirm the diagnosis of supra- and infratentorial brain abscesses and left eye endophthalmitis with a subretinal abscess (Fig. 2).

Magnetic resonance cholangiopancreatography was performed to rule out malignancy and hepatic lesions; however, it only showed a cirrhotic liver with portal hypertension without any focal lesions. However, an incidental finding of bilateral lower-lobe pulmonary nodules was noted, for which a dedicated chest CT was planned.

**Fig. 3:**
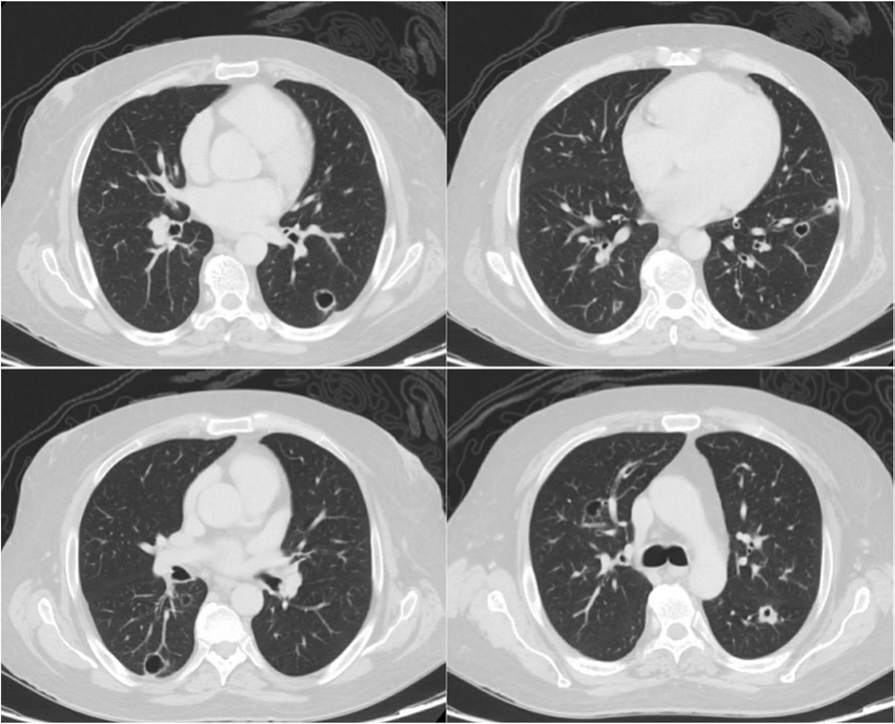
Axial sections of a contrast-enhanced CT scan of the chest show multiple bilateral cystic and cavitary lung lesions, predominantly involving the lower lobes. A cavitary nodule is seen in the superior segment of the left lower lobe showing thickened wall (*upper left image*)

High-resolution chest CT revealed multiple small bilateral cystic and cavitary lung lesions (Fig. 3).

The patient underwent further investigations, including blood culture, bronchoalveolar lavage culture, AFB of three samples from saliva, QuantiFERON TB gold test, HIV tests, and syphilis serology. However, all tests had negative results.

An ophthalmic examination shown in (Table 1) revealed a subretinal abscess in the right eye. The patient was initially suspected to have endogenous endophthalmitis and was started on intravitreal vancomycin, ceftazidime, and voriconazole. A few days later, the patient’s left eye failed to improve, and as a result, evisceration of the left eye was decided.

Microbiological studies were performed on the left eye specimen; Ziehl–Neelsen (ZN) staining was negative; however, the modified ZN stain was positive. Partial acid-fast bacteria (AFB) stained positive.

Culture of the left eye swab done in Blood agar and MacConkey agar confirmed the diagnosis of *Nocardia farcinica* infection. Identification was performed using MALDI-TOF and sensitivity using the manual E-test (Table 2).

**Table 2:** Patient minimum inhibitory concentration with CLSI M24-A2 reference criteria for sensitivity

Antibiotic	Patient MIC^a^	CLSI M24-A2 MIC Susceptible	CLSI M24-A2 MIC Intermediate	CLSI M24-A2 MIC Resistant
Trimethoprim-sulfamethoxazole	0.5 μg/mL	≤ 2/38	-	≥ 4/76
Linezolid	1.0 μg/mL	≤ 8	-	-
Amoxicillin clavulanate	2.0 μg/ml	≤ 8/4	16/8	≥ 32/16
Ciprofloxacin	0.047 μg/mL	≤ 1	2	≥ 4
Amikacin	0.5 μg/mL	≤ 8	-	≥ 16
Ceftriaxone	2.0 μg/mL	≤ 8	16–32	≥ 64
Imipenem	0.5 μg/mL	≤ 4	8	≥ 16
Doxycycline	4 μg/mL	≤ 1	2–4	≥ 8
Minocycline	1.0 μg/mL	≤ 1	2	≥ 8
Clarithromycin	0.75 μg/mL	≤ 2	4	≥ 8

^a^ *MIC* Minimum inhibitory concentration

Histopathological examination revealed dense histiocytic and neutrophilic infiltrates consistent with an abscess (acute endophthalmitis). Special staining including Grocott’s methenamine silver, and periodic acid-Schiff staining yielded negative results, while Acid-fast bacteria (AFB) satin was partially positive.

When disseminated nocardiosis was confirmed, the patient was started on Nocardia treatment (imipenem 500 mg every 8 h IV, trimethoprim/sulfamethoxazole 255 mg every 6 h, and amikacin 15 mg/kg once daily). Within a few days, the patient’s condition improved with complete resolution of the subretinal abscess in the right eye. Follow-up ophthalmic examination during treatment is shown in (Table 3).

**Table 3:** Ophthalmic examination during treatment

*Ophthalmic examination*	Right	Left
External Exam	Normal	Eviscerated
Visual acuity	Counting fingers	
Slit Lamp Exam		
Cornea	Clear	
Anterior chamber	Deep and quite	
Iris	Medically dilated	
Lense	Clear	
Vitreous	Clear	
Fundus exam		
Macula	Resolved macular abscess. Retinal pigment epithelium (RPE) changes around the inferotemporal arcade	

**Fig. 4:**
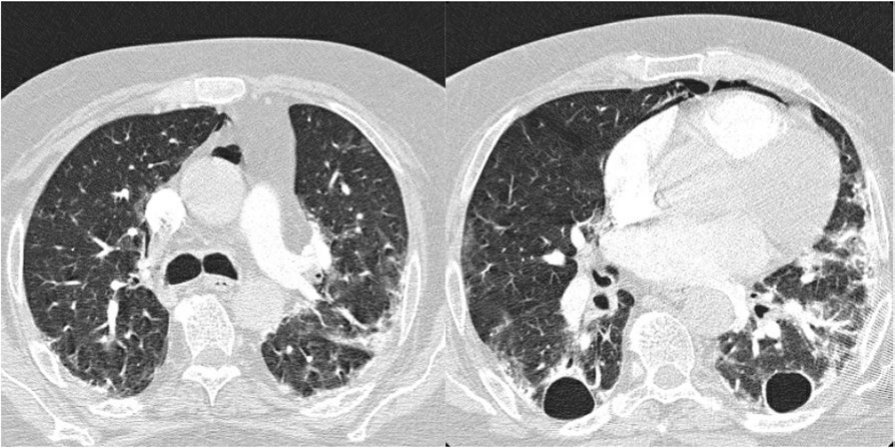
Axial sections of CT angiography of the pulmonary arteries, performed a month after the previous chest CT (Fig. 3), show progression of bilateral cavitary lung lesions and more evident traction bronchiectasis. It also shows development of bilateral peripheral patchy ground glass opacities and compressive atelectasis. Note is also made of pneumomediastinum

Follow-up magnetic resonance imaging of the brain also showed interval regression in the size of multiple peripherally located bilateral ring-enhancing lesions. However, CT angiography of the chest, which was performed to rule out pulmonary embolism a month after the previous chest CT, revealed progression of multiple bilateral cavitary lung lesions with the development of pneumomediastinum (Fig. 4).

During hospital stay, the patient developed acute pancreatitis and acute kidney injury, which were primarily treated with hydration and pain management.

The patient received imipenem, amikacin, trimethoprim/sulfamethoxazole, and neomycin for 6 weeks. The patient was maintained on ocular prednisolone and cyclopentolate, as recommended by an ophthalmologist.

The patient’s hospital course was complicated by his aggressive and advanced condition. He developed septic shock and died of cardiac arrest in the intensive care unit.

## Discussion

In most cases, disseminated nocardiosis is transmitted from the lungs to the central nervous system, skin, and soft tissues. This is usually observed in severely immunocompromised patients (patients with AIDS and transplant recipients) [2].

We performed a literature review by searching the MEDLINE database using keywords, including “nocardia,” “nocardiosis,” “alcohol,” and “cirrhosis,” and we found that this is an extremity rare case of disseminated nocardiosis in a patient with a medical background of alcoholic liver cirrhosis.

Torres et al. and Budzik et al. reviewed 53 and 67 cases of *Nocardia farcinica*, respectively. According to Torres et al., 85% of patients have predisposing factors, and Budzik et al. further investigated these factors. Most patients (61.2%) were immunosuppressed (on chemotherapy or steroids), 17% were solid organ transplant recipients, 13% had chronic obstructive pulmonary disease, 9% had diabetes mellitus, 7.5% were infected with human immunodeficiency virus, another 7.5% had solid neoplasms, and the last 3% had alcoholism [7, 8]. Budzik et al. reviewed 3 patients who were alcoholics that were infected with N. farcinica. Two out of the three patients had another underlying immunosuppressive condition, including renal transplant and diabetes. The third patient was exclusively alcoholic who had an infection involving the CNS, spine, and the psoas muscle [8].

(Table 4) shows the percentage of organ involvement in *N. farcinica* infection in both studies, where the lung was the most affected organ in both sets of patients, and the eye was infected in only two out of both sets of patients. A comparison of the antibiotic susceptibility of *N. farcinica* isolated in the cases reviewed by Torres et al. and those isolated in our case demonstrates that most patients were sensitive to trimethoprim-sulfamethoxazole, amikacin, and imipenem [7, 8].

**Table 4:** Organ involvement in *N. farcinica* infection

Organ involvement	Torres et al. *n* = 53 (%)	Budzik et al. *n* = 67 (%)
Lung	23 (43%)	40 (59%)
Brain	16 (30%)	22 (32%)
Wound infections	8 (15%)	12 (17.9%)
Spine	2 (3%)	—
Kidney	4 (8%)	1 (1.5%)
Lymphatic	1 (1.5%)	—

The recommendation is for immunocompetent patients infected with *N. farcinica* who are susceptible to trimethoprim-sulfamethoxazole, moxifloxacin, amikacin, minocycline, linezolid, ciprofloxacin, and imipenem-cilastatin to be treated for at least 6 months, and the period could increase to at least 12 months if the central nervous system is involved [9–12]. In our case, the patient was started on imipenem, trimethoprim/sulfamethoxazole, and amikacin, and within a few days, the patient’s condition remarkably improved with complete resolution of the subretinal abscess in the right eye. However, owing to the patient’s advanced and aggressive disease, the he developed septic shock and died in the intensive care unit.

Immunocompromised patients with nocardial infections often exhibit cell-mediated abnormalities [13, 14].

Liver cirrhosis is an atypical immunosuppressive condition associated with immune dysfunction regardless of the cause. This leads to alterations in the hemostatic role of the liver and disrupts the innate and acquired immunity by causing defects in both local and systemic immunity leading to a high mortality rate (30%) and an increase in systemic inflammation and immunodeficiency [15].

## Conclusion

Although the patient’s condition initially improved with imipenem, trimethoprim/sulfamethoxazole, and amikacin, the patient’s hospitalization course was complicated and led to death. Early detection of nocardial infection in patients with typical or atypical immunosuppressive conditions may improve overall mortality and morbidity.

## Data Availability

The datasets used and/or analysed during the current study available from the corresponding author on reasonable request.
